# Nonlinear Topological Photonics: Capturing Nonlinear Dynamics and Optical Thermodynamics

**DOI:** 10.1021/acsphotonics.4c02430

**Published:** 2025-04-29

**Authors:** Stephan Wong, Alexander Cerjan, Konstantinos G. Makris, Mercedeh Khajavikhan, Demetrios Christodoulides, Sang Soon Oh

**Affiliations:** † Center for Integrated Nanotechnologies, 1105 Sandia National Laboratories, Albuquerque, New Mexico 87185, United States; ‡ ICTP, Department of Physics, University of Crete, 71003 Heraklion, Greece; ¶ Ming Hsieh Department of Electrical and Computer Engineering, University of Southern California, Los Angeles, California 90089, United States; § School of Physics and Astronomy, Cardiff University, Cardiff CF24 3AA, U.K.

**Keywords:** Topological photonics, optical nonlinearity, nonlinear dynamics, machine learning, spectral localizer, pseudospectrum

## Abstract

Combining multiple optical resonators or engineering dispersion of complex media has provided an effective method for demonstrating topological physics controlling photons in unprecedented ways such as unidirectional light propagation and spatially localized modes between an interface or on a corner. Further, adding nonlinear responses to those topological photonic systems has enabled achieving diverse phases of photons in both space and time, allowing for more functionalities in photonic devices that provide a new playground for studying dynamic features of nonlinear topological systems. However, most methods for describing nonlinear topological photonic systems rely on linear topological theories, making it challenging to accurately characterize the topology of nonlinear systems. Thus, substantial efforts have focused on rigorously describing nonlinear topological phases and developing effective tools to analyze nonlinear topological effects. Meanwhile, coupled multimode optical waveguides with nonlinear dynamic responses provide an excellent platform for the statistical description of photons, opening a new paradigm called “optical thermodynamics”. This review will introduce the basic concepts of nonlinear topological photonics and the recent development of theoretical approaches focusing on data-driven approaches for creating phase diagrams as well as the spectral localizer framework and the pseudospectrum method for understanding optical nonlinearities in topological systems. In addition, the new concept of optical thermodynamics will be introduced with some recent theoretical works.

## Graphical abstract

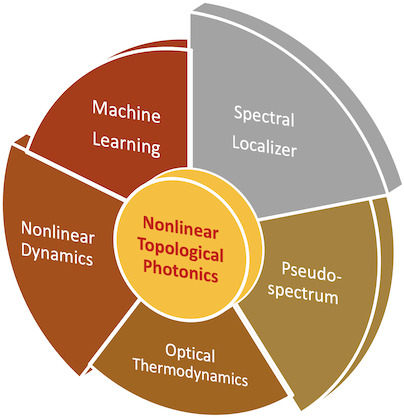

## 1 Introduction

Topological photonics started by applying topological band theory developed in condensed matter to periodic photonic structures with linear optical responses such as an array of optical resonators, photonic crystals, and metamaterials.^1^ Then, the scope of topological photonics has been extended by including nonlinearities such as saturable optical gain and Kerr nonlinearities in the constituting materials.^2–5^ For the past decade, the research on nonlinear topological photonics has proliferated, embracing wider areas not previously covered in linear topological photonics, e.g., spatiotemporal dynamics,^6^ optical bistabilities,^7,8^ and lasing^9^ in both Kerr nonlinear and saturated gain settings as shown in Figure 1(a–c).

**Figure 1:**
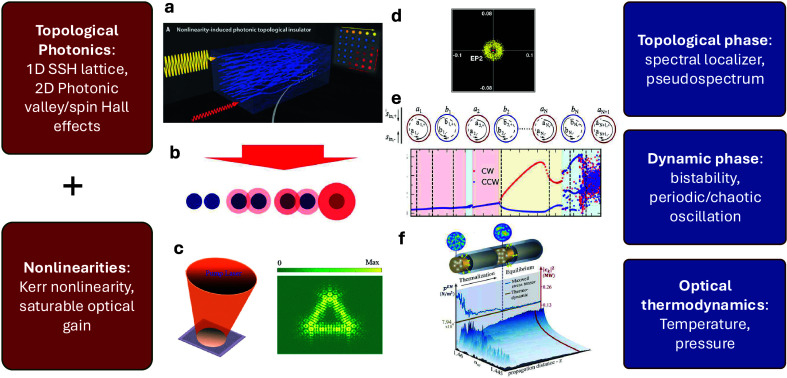
Schematics of nonlinear topological photonic structures. (a) A nonlinearity-induced photonic topological insulator. Adapted with permission from ref 17. Copyright 2020 The American Association for the Advancement of Science. (b) A Su-Schrieffer-Heeger lattice with Kerr nonlinearity. Adapted with permission from ref 18. Copyright 2018 American Physical Society. (c) An optically pumped topological laser with a kagome lattice. Adapted with permission from ref 19. Copyright 2020 American Chemical Society (d) A pseudospectrum for a finite SSH lattice. Adapted with permission from ref 13. Copyright 2022 American Physical Society. (e) Dynamic phases in a nonlinear SSH ring resonator. Adapted with permission from ref 8. Copyright 2024 American Physical Society. (f) Thermalization in a multimode optical waveguide. Adapted with permission from ref 20. Copyright 2023 American Physical Society.

Optical nonlinearities give rise to a wide range of effects, including self- and cross-phase modulation, two- and multiphoton absorption, second harmonic generation, and four-wave mixing. These phenomena are primarily governed by the material’s nonlinear response and the specifics of the pump-probe configuration, such as phase-matching conditions and energy conservation. Traditionally, they are studied in spatially uniform media, such as nonlinear crystals or single optical resonators. In contrast, investigating nonlinear effects in extended structures, such as two-dimensional (2D) arrays of optical resonators, photonic crystals, and metasurfaces, introduces richer physics and opens pathways toward practical applications in topological photonics (Figure 1(b)­(c) and (e)). However, understanding and analyzing experimental and numerical results remain challenging, as conventional linear topological band theory does not apply. To address this, various theoretical and computational approaches have been developed, including data-driven methods,^10^ the spectral localizer framework,^11,12^ and the pseudospectra method^13^ (Figure 1(d)), which have been successfully applied across different nonlinear optical systems.

Recently, it has been proposed to use coupled nonlinear optical waveguides as a platform for optical thermodynamics.^14–16^ The complexity and increased number of states of nonlinearly coupled multimode systems, for instance, a multimode waveguide shown in Figure 1(f), require thermodynamical descriptions. This brings us to a new regime of thermodynamics where macroscopic parameters can be defined for the ensemble of photons in the coupled nonlinear systems.

In this review, we mainly focus on new theoretical and numerical tools to analyze nonlinear optical topological systems and review the most recent advances. First, we will look at a way of drawing phase diagrams of nonlinear photonic topological insulators with optical gain and loss using a data-driven approach. Second, we introduce the spectral localizer to define topological invariants in a topological photonic system with a finite size with an example with Kerr optical nonlinearity. Third, we look into a pseudospectral method applied to a photonic topological system with optical nonlinearity. Finally, we introduce optical thermodynamics in nonlinear coupled multimode waveguide systems. Here, we begin with a brief overview of the fundamental principles of nonlinear topological photonics. For readers interested in a more comprehensive discussion of the physical effects and underlying nonlinearities, we refer to existing reviews that cover these topics in greater detail.^2,21^

## 2 Phase Diagrams for Topological Phases and Dynamic Phases

The interplay between the topological system and nonlinearities gives rise to rich dynamical behaviors of the mode, leading to a plethora of novel topological phenomena with no counterpart in the linear regime. As such, the dynamics plays a significant role in the study of nonlinear topological insulators, which is often captured by the nonlinear Schrödinger equation

1 $$i\frac{d}{dt}\psi \left(t\right)=\left[H+H_{NL}\left(\psi \right)\right]\psi \left(t\right)$$

where *H* is the linear part of lattice Hamiltonian with open boundary conditions, and *H*_NL_ is the nonlinear part with a state-dependency ψ­(*t*) that is mainly of Kerr-type |ψ­(*t*)|^2^ or saturable-type 1/(1 + |ψ­(*t*)|^2^) in photonic systems. In particular, by generalizing linear topological insulators to nonlinear topological insulators, the nonlinear terms introduce fundamental components and challenges to nonlinear topological insulators, such as the need to take into account the local feature of the nonlinearities and the temporal dynamics of the modes. Overall, the study of topology and the existence of topological modes is no longer confined solely to the frequency-domain within topological band theory. Instead the nonlinearities enforce a paradigm shift, requiring the study to incorporate real-space and time-domain information.

### 2.1 Temporal Stability

While nontrivial topology in a nonlinear system guarantees the existence and robustness of a nonlinear topological mode against structural defect of the system, the topologically protected mode may not survive over time against disorders due to temporal instabilities.^22,25–27^ In particular, using linear stability analysis, the temporal stability is analyzed by considering small perturbations of the form (*u* + *iv*)*e* ^*iωt*^ around a solution ψ^(0)^(*t*) of the nonlinear Schrödinger equation [eq 1]. The ansatz for the perturbed nonlinear solution, ψ­(*t*) = ψ^(0)^(*t*) + (*u* + *iv*)*e* ^*iωt*^, is then plugged into eq 1, and solved for ω. The temporal stability is then determined by looking at the sign of Im­(ω), where unstable and stable behaviors are characterized by the exponential decay (Im­(ω) > 0) or growth (Im­(ω) < 0) of the perturbations. Therefore, the obtained nonlinear topological mode may not be temporally robust as small fluctuation may grow over time. For example, using a PT -symmetric Su-Schrieffer-Heeger (SSH) lattice with an imaginary gauge field, a topological mode is guaranteed in the unbroken PT phase.^28,29^ However, incorporating the nonlinearities in the gain and loss renders the topological mode temporally unstable for some gain parameters.^22^ By scanning over the parameter space, stability diagrams of the topological mode can be calculated, giving information about the practical existence of the topological mode over time, as shown in Figure 2(a).

**Figure 2:**
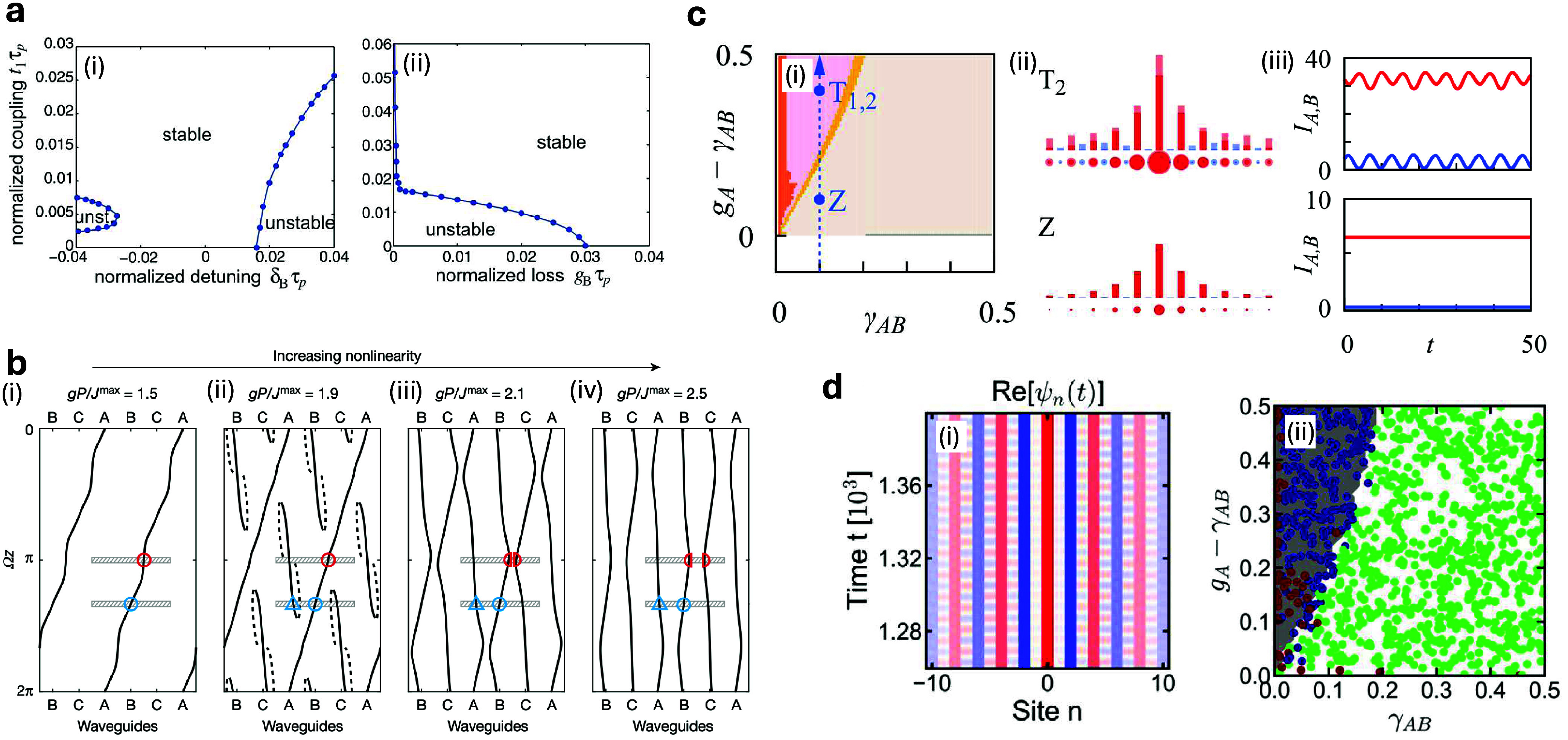
(a) Stability diagram for the zero mode in a PT -symmetric SSH lattice with imaginary gauge field for (i) the parameter space composed of the coupling constant and detuning parameters and (ii) the coupling constant and loss parameter. “Stable” and “unstable (or unst)” mean the parameter spaces which correspond to the stable and unstable phases, respectively. Adapted with permission from ref 22. Copyright 2018 John Wiley and Sons. (b) Mechanism for trivial and nontrivial quantized transport in a Thouless system. The solid (dashed) lines correspond to the soliton’s center of mass being temporally stable (unstable). The blue and red markers represent specific soliton positions undergoing different bifurcations. Adapted with permission from ref 23. Copyright 2021 Springer Nature. (c) Topological lasing regime for a SSH array with saturable gain, and the profile and dynamics of the topological modes. Adapted with permission from ref 24. Copyright 2018 IOP Publishing Ltd. (d) Topological phase diagram obtained from a data-driven bottom-up representation classification, reproducing the theoretical prediction in (c). The blue, green, and red dots are the identified dynamical phases corresponding to the oscillating modes, zero modes, and transient regime modes, respectively. Adapted with permission from ref 10. Copyright 2023 Springer Nature.

### 2.2 Bifurcation-Related Topological Phase Transition

Similar to topological invariants in linear topological insulators, the temporal stability also gives us information on the existence of a nonlinear topological mode. While in the linear regime, the change in the number of topological modes is related to a gap closing as dictated by topological band theory,^30^ this change can be paralleled in the nonlinear regime by the change of temporal stability happening at bifurcation points. Therefore, despite leading to potential temporal instabilities, nonlinearities offer new mechanisms for topological phase transitions happening at bifurcation points. For example, in a Thouless pump system, it has been shown that nonlinearities can act as a way to achieve quantized transport via the formation of a soliton, with a nonlinearly induced topological phase transition occurring at the bifurcation point.^23,31^ In particular, at low power, depending on the Chern number of the participating bands constituting the soliton, the soliton’s center of mass is displaced by an integer number of unit cells, demonstrating the nontrivial topology of the soliton [see Figure 2(b) for up to *gP*/*J* ^max^ = 1.9]. When the power is increased, a pitchfork bifurcation occurs, resulting in the splitting of the path of the soliton’s center of mass. This splitting makes the soliton to return to the site from which it started at the beginning of the cycle, leading to a trivial displacement and thus trivial topology [see Figure 2(b) for *gP*/*J* ^max^ = 2.1]. As such, there is a topological phase transition taking place at a symmetry-breaking bifurcation with increasing nonlinear parameter.

From a symmetry perspective, the topological modes can be also classified with respect to their excitation spectrum, featuring robust spectral signatures pinned to symmetry-protected positions, which can change only in further phase transitions. In particular, the excitation spectrum of a topological mode can be obtained from the excitation Hamiltonian H during the linear stability analysis. The excitation Hamiltonian H is identified as the Hamiltonian of the system when the governing equation is rewritten in the basis of the perturbation

2 $$i\frac{d}{dt}\mathrm{\psi ̃}\left(t\right)=H\left(\psi^{\left(0\right)}\right)\mathrm{\psi ̃}\left(t\right)$$

with ψ^(0)^ the nonlinear solution over which linear stability is realized, and ψ̃(*t*) = (*u*,*v*). As such, the robust spectral signature characterizing the topological modes comes from the symmetry of the excitation Hamiltonian H and can only be changed at a bifurcation point, which can be associated with a topological phase transition. The topological aspect from the excitation spectrum has been illustrated and developed in refs 24 and 32 with one-dimensional topological laser arrays with saturable gain and charge-conjugation symmetry. These charge-conjugation symmetric systems have been shown to support stable symmetry-protected zero modes as well as symmetry-protected power oscillations with no counterpart in the linear case [see Figure 2(c) bottom panels]. The analysis of the excitation spectrum of these modes shows spectral signatures that are pinned to symmetry-protected positions, in analogy to Majorana zero modes in Fermionic systems with charge-conjugation symmetry. In this regard, these features uncover topological phase transitions in which zero modes and oscillating states interchange their temporal stability at a bifurcation point, resulting in the topological phase diagram shown in Figure 2(c).

### 2.3 Machine Learning Approach

With the advent of machine learning methods and its success in identifying topological phases in linear systems,^33–39^ machine learning approaches show promising results in identifying topological phases in nonlinear systems. In particular, assuming the topological nature of the states can be ascertained by their spatial and dynamical characteristics, a data-driven approach can be used to identify the topological phases and their bifurcation-related topological phase transition over a given parameter space. The data-driven approach can thus be used as a preliminary tool for a rapid exploration of the parameter space to find the topological modes, which are often difficult to find analytically and require advanced knowledge on the complex nonlinear system, before being complemented by a more thorough analysis of their excitation spectra. For example, a bottom-up representation classification method^10^ has been used to classify the topological lasing modes of an SSH lattice with a domain wall and saturable gain, based on their distinct nonlinear regimes. The general idea consists of constructing a library L , made of the different dynamical regimes of interest, and then identify the simulated system’s dynamics ψ­(*t*) to a regime in the library. Given the library is written as

3 $$L=\left\{\Phi_{1},...,\Phi_{J}\right\}={\left\{\phi_{j,i}\right\}}_{j=1,...,J,i=1,...,D}$$

with *J* the number of regimes, Φ_*j*_’s the bases representing the dynamical regime *j*, and ϕ_*j*,*i*_’s the corresponding basis states, the system’s dynamics ψ­(*t*) is assumed to be written as a linear combination of the different dynamical regimes in L as

4 $$\psi \left(t\right)\approx \overset{J}{\underset{j=1}{\sum }}\overset{D}{\underset{i=1}{\sum }}\phi_{j,i}\beta_{j,i}\left(t\right)=\overset{J}{\underset{j=1}{\sum }}\Phi_{j}\beta_{j}\left(t\right)$$

where β_*j*,*i*_ are the weighted coefficients. The correct regime j* is then identified as the corresponding subspace in the library L closest to the measurement in the *L* ^2^-norm sense. With the basis in L generated using a time-augmented dynamical mode decomposition (aDMD) method,^40^ to take into account both the spatial and temporal behaviors of the system’s dynamics, the data-driven approach has been able to correctly reproduce the phase diagram in the SSH lattice with saturable gain, as shown in Figure 2(d) (see also Figure 2(c) as the analytical prediction). With the capability of clustering similar behavior, a reverse engineering procedure holds the potential to find novel topological lasing modes that may have been overlooked in other approaches. As such, in this context of nonlinear systems and temporal dynamics, machine learning methods provide unique advantages, such as rapid exploration of the parameter space and valuable insight into the topological classification without requiring advanced prior knowledge or case-by-case parameter calculations.

However, there are potential challenges in using machine learning methods. Setting hyperparameters appropriately is crucial to avoid over- or under-estimating the results. For instance, selecting a threshold that is too high or too low when determining the equivalence of dynamical phases can lead to undesirable outcomes: a threshold set too high may result in identifying too many distinct topological phases, while a threshold set too low may fail to distinguish between phases altogether.

### 2.4 Future Directions

The studies presented here on nonlinear topological insulators show great potential to tackle the classification problem from the dynamical point of view. While machine learning approaches can accelerate the exploration of parameter space, offering rapid insights, the topological classification problem may also benefit greatly from employing tools from the nonlinear physics such as numerical continuation.^41,42^ Numerical continuation will then help to get information in a more systematic way about the solution path of the nonlinear solutions and their temporal stability in a given parameter space, allowing to explore the topological phase transition in nonlinear systems with different shapes and (unitary and nonunitary) symmetries.

## 3 Classifying Topology in Nonlinear Materials Using the Spectral Localizer Framework

One of the fundamental challenges in identifying topological phenomena in nonlinear photonic systems is the local nature of both the nonlinear material responses as well as many forms of photonic excitations, such as solitons.^17,23,31,43–46^ As such, topological band theory is poorly suited to classifying nonlinearly induced topological phase transitions in photonic systems, as a local excitation, in tandem with a local nonlinear response, yields a spatially nonuniform system that lacks the translational symmetry required to define a band structure. Thus, determining the topological invariants associated with nonlinearities in photonic systems typically demands the use of *local topological markers*,^47,48^ which provide a method of identifying a system’s topology at a specified location ***x*** = (*x*_1_, ..., *x*_*d*_) for a *d*-dimensional system and energy *E*, as opposed to standard topological invariants defined using a system’s Bloch eigenstates,^1^ which are global properties of a material at a given *E* in a bulk spectral gap.

In particular, the spectral localizer framework^12,49–51^ has emerged as a promising approach for classifying topological phase transitions induced by a nonlinear system’s local excitations.^52,53^ This framework uses a position-space description of a finite system and provides both a suite of local markers for a broad range of classes of material topology as well as a local measure of topological protection. Indeed, this second feature of yielding a provably local quantitative bound for a topologcial phase’s robustness distinguishes the spectral localizer framework from other theories of local markers, which instead appeal to a system’s bulk spectral gap for defining topological protection. The mathematical origins of this framework are in C*-algebras, not vector bundles, and so its formulas cannot be easily related to the invariants of topological band theory. Overall, the spectral localizer framework can classify the topology of all of the ten Altland-Zirnbauer classes^54–56^ in any physical dimension, as well as some forms of crystalline topology,^57^ non-Hermitian topology,^58,59^ and Weyl semimetals;^60^ it is also possible to identify Chern materials in the presence of non-Hermiticity.^61,62^ However, this section will only address Chern materials (2D class A)^52^ and materials described by winding numbers (1D class AIII),^53^ the classes of topology that prior studies have considered photonic nonlinearities in.

### 3.1 Introduction to the Spectral Localizer Framework

The spectral localizer framework combines a finite system’s Hamiltonian *H* (i.e., with open boundaries) with its position operators ***X*** = (*X*_1_, ..., *X*_*d*_) at a given choice of position and energy (***x***, *E*) using an irreducible Clifford representation to form a composite operator called the *spectral localizer*. For a 2D system, the position operators are *X* and *Y*, and the Pauli matrices σ_*x*,*y*,*z*_ can be chosen as the Clifford representation, yielding a 2D spectral localizer

5 $$\begin{array}{l} L_{\left(x,E\right)}^{\left(2D\right)}\left(X,Y,H\right)\\ =\kappa \left(X-x1\right)⊗\sigma_{x}+\kappa \left(Y-y1\right)⊗\sigma_{y}+\left(H-E1\right)⊗\sigma_{z}\\ =\left(\begin{array}{cc} H-E1 & \kappa \left(X-x1\right)-i\kappa \left(Y-y1\right)\\ \kappa \left(X-x1\right)+i\kappa \left(Y-y1\right) & \left(H-E1\right) \end{array}\right) \end{array}$$

Here, **1** is the identity and κ ≥ 0 is a scaling coefficient that both ensures consistent units, i.e., κ has units of energy divided by length, as well as balances the *spectral weight* of the Hamiltonian relative to the position operators. In other words, κ is chosen so that the eigenvalues of *L*_(**x**,*E*)_ are similarly sensitive to changes in *H* – *E* **1** and changes in *X* – *x* **1** and *Y* – *y* **1**, either because the choice of (***x***, *E*) is shifted or because the system is perturbed *H* → *H* + *δH*. Although the introduction of such a hyper-parameter is typically unappealing, one can prove that for bounded Hamiltonians with bulk spectral gaps, a valid range of κ always exists.^50,51^ Moreover, in practice a useful range of κ typically spans over 2 orders of magnitude even for modestly sized systems and contains κ ∼ *E*_gap_/*l*, where *E*_gap_ is the relevant bulk spectral gap and *l* is the shortest length of the finite system.

A local Chern marker can be defined through eq 5 as

6 $$C_{\left(x,E\right)}^{L}\left(X,Y,H\right)=\frac{1}{2}sig\left[L_{\left(x,E\right)}^{\left(2D\right)}\left(X,Y,H\right)\right]\in Z$$

Here, sig­[*M*] is the signature of the Hermitian matrix *M*, which is the difference between its number of positive eigenvalues and its number of negative eigenvalues. Given this definition, the local Chern marker is guaranteed to be an integer for any choice of (***x***, *E*). For a semi-infinite crystalline material with a bulk band gap, one can prove that *C*_(**x**,*E*)_^L^ is equal in magnitude to the standard Chern number defined by topological band theory,^50^ while the sign ambiguity can be traced back to the choice of Clifford representation used in the spectral localizer.

Intuitively, the local Chern number can be understood through dimensional reduction and Bott periodicity, facilitated by the Clifford representation. For any choice of (***x***, *E*), *L*_(**x**,*E*)_^(2D)^ is Hermitian, and can be viewed as the Hamiltonian of some fictitious zero-dimensional molecule. If *L*_(**x**,*E*)_^(2D)^ is invertible, i.e., none of its eigenvalues are 0, its signature is an invariant of homotopy: any 0D system with an invertible Hamiltonian can be connected to any other by a path of invertible Hermitian matrices so long as they have the same signature. In other words, if *L*_(**x**,*E*)_^(2D)^ is invertible, it represents a molecule with an energy gap at *E* = 0, and it can be connected to any other molecule with the same signature without closing this gap; indeed, (1/2)­sig­[*H*] is the (0th) Chern number for a 0D system (0D class A).^47^ Finally, the use of the Clifford representation in the spectral localizer ensures that the zeroth Chern number of the fictitious 0D molecule is the same as the (first) Chern number of the underlying 2D system.^12,49,50^

This intuitive argument for understanding the spectral localizer also reveals its inherent measure of topological protection. For the topology of the fictitious 0D molecule to change due to some perturbation *δH*, one of the eigenvalues of *L*_(***x***,*E*)_^(2D)^(***X***,*H* + *δH*) must first become 0 as the strength of the perturbation is increased. However, as the spectral localizer is Hermitian, its eigenvalues must move continuously, and the distance they can move is bounded by the largest singular value of the perturbation ∥*δH*∥, i.e., the *L* ^2^ matrix norm. Thus, the eigenvalue of *L*_(**x**,*E*)_^(2D)^ closest to zero

7 $$\mu_{\left(x,E\right)}\left(X,H\right)=\min\left[|spec\left(L_{\left(x,E\right)}\left(X,H\right)\right)|\right]$$

defines a measure of topological protection: any perturbation with strength ∥*δH*∥ < μ_(***x***,*E*)_(***X***, *H*) cannot change the system’s local topology at (***x***, *E*). Here, spec­[*M*] is the spectrum of *M*. Finally, as the spectral localizer has units of energy, so does μ_(***x***,*E*)_, and thus it can be understood as a *local gap*.

To identify topology in nonlinear 1D systems with chiral symmetry Π, with Π*H* = – *H*Π, one instead uses the *symmetry reduced spectral localizer* ^12,63^

8 $${\mathrm{L̃}}_{\left(x,E\right)}^{\left(1D\right)}\left(X,H\right)=\left[\kappa \left(X-x1\right)-i\left(H-E1\right)\right]\Pi$$

Note that for odd-dimensional systems, there is a basis in which the full spectral localizer is off-block diagonal, and only a single one of these blocks is used in the definition of the local topological markers. For example, ${\mathrm{L̃}}_{\left(x,E\right)}^{\left(1D\right)}$ is the upper-right block of *L*_(*x*,*E*)_^(1*D*)^ = κ­(*X*-*x*1)⊗σ_*x*_ + κ­(*H*-*E*1)⊗σ_*y*_ multiplied by Π. By multiplying by the chiral symmetry operator, ${\mathrm{L̃}}_{\left(x,0\right)}^{\left(1D\right)}$ is Hermitian, and can be used to define the local winding number

9 $$\nu_{x}^{L}\left(X,H\right)=\frac{1}{2}sig\left[{\mathrm{L̃}}_{\left(x,0\right)}^{\left(1D\right)}\left(X,H\right)\right]\in Z$$

Finally, the topological protection of 1D systems is still given by the local gap, eq 7.

### 3.2 Classifying Nonlinear Systems

As the spectral localizer framework does not make any assumptions about the spatial structure of the system being considered, or even whether its underlying Hamiltonian has a spectral gap, it can identify topology in aperiodic,^64^ gapless,^11,63^ and nonlinear systems^52,53^ without alteration. In particular, to identify nonlinearly induced topology, all that is needed is the nonlinear Hamiltonian *H*(**ψ**) and the system’s current occupation **ψ**; these quantities can then be directly inserted into the spectral localizer as *L*_(***x***,*E*)_(***X***, *H*(**ψ**)), and then the local markers and associated local gap can be determined as normal through eq 6, eq 9, and eq 7.

For example, consider a nonlinear 2D Haldane lattice,^52^ whose linear response *H*_0_ is given by the Haldane model^65^ and with an on-site Kerr-type nonlinearity parametrized by *g*,

10 $${\left[H\left(\psi \right)\right]}_{mn}={\left[H_{0}\right]}_{mn}+g{\left|\psi_{n}\right|}^{2}\delta_{mn}$$

Here, δ_*mn*_ is the Kronecker delta function. In the absence of any occupation, **ψ** = **0**, the linear system is in a topological phase and can be classified using the local Chern marker, shown in Figure 3(a–c). However, if the system is occupied by a self-consistent solution with an energy within the linear system’s topological band gap, the system undergoes a local nonlinear topological phase transition where the occupation is strongest, shown in Figure 3(d–f). Note, although this particular example is using a self-consistent solution of the nonlinear Hamiltonian, the local topology of any occupation can be classified using the spectral localizer framework, yielding a rigorous approach to classifying topological dynamics for systems with evolving occupations **ψ**(*t*).

**Figure 3:**
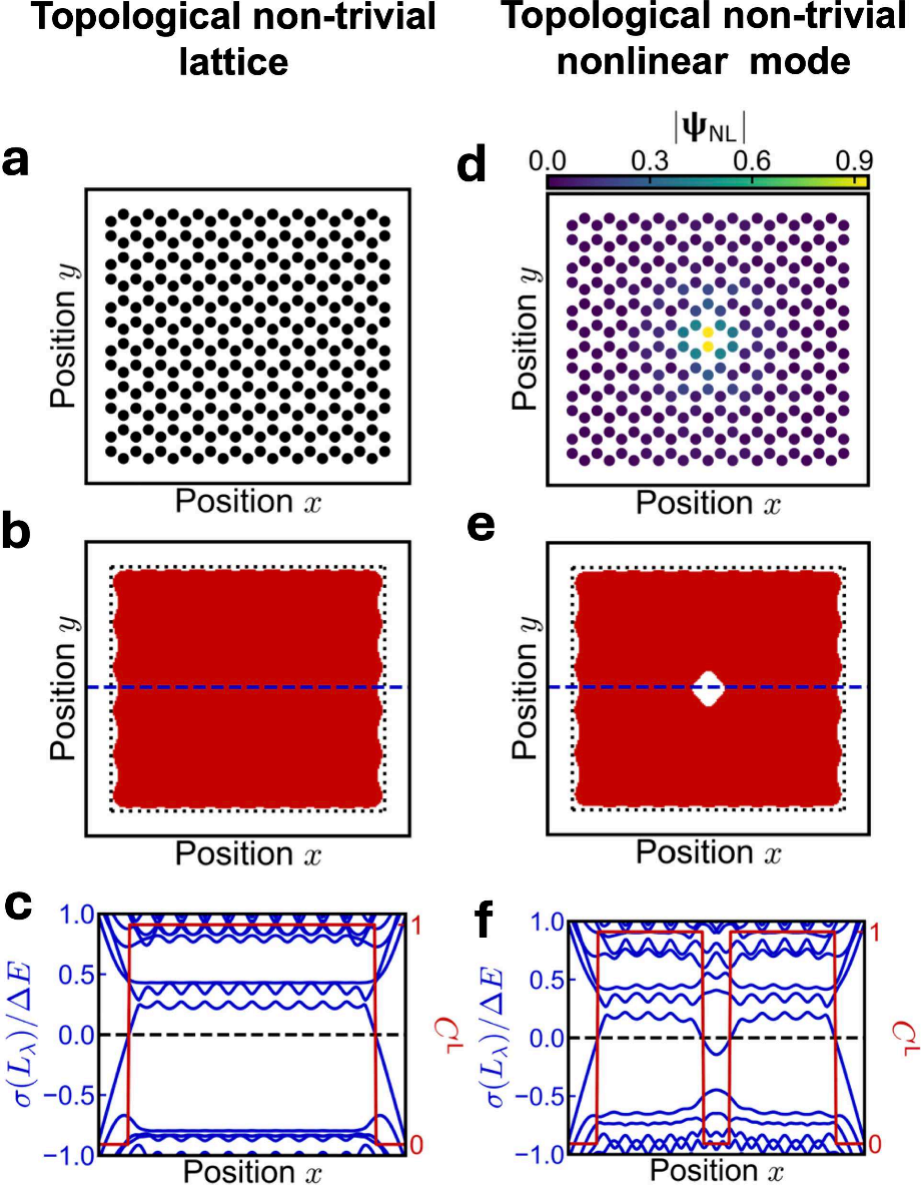
Classification of nonlinear topology using the spectral localizer framework. (a) Diagram of a finite portion of a linear Haldane lattice. (b) Local Chern marker as a function of probe position ***x*** for *E* the center of the topological band gap with width Δ*E* for *k* = 2­[*t*/*a*], where *t* is the linear nearest neighbor coupling and *a* is the lattice constant. (c) Spectrum of the spectral localizer across the center of the lattice. (d) Distribution of a self-consistent solution to the nonlinear Haldane lattice with eigenenergy in the topological band gap. (e, f) Similar to (b, c), except for the occupied nonlinear lattice. Adapted with permission from ref 52. Copyright 2023 American Physical Society.

In addition, the local measure of topological protection provided by the spectral localizer framework acquires an additional physical meaning in nonlinear systems: it guarantees the continuing existence of a self-consistent solution against system perturbations. Specifically, if **ψ** is a self-consistent solution to a nonlinear Hamiltonian that induces a local topological phase change, there is a location within the induced topological domain where the local gap is maximized μ_NL_ = μ_(***x***,*E*)_(***X***, *H*). In general, there is no guarantee that a given self-consistent nonlinear solution survives the addition of any perturbation. However, if the self-consistent solution is topologically protected by μ_NL_, a perturbation cannot cause the solution curve of **ψ** to terminate until ∥*δH*∥ ≥ μ_NL_, as the perturbation would otherwise change the occupied system’s local topology. Indeed, ref 52 numerically verified this prediction over an ensemble of different disorder configurations; see Figure 4.

**Figure 4:**
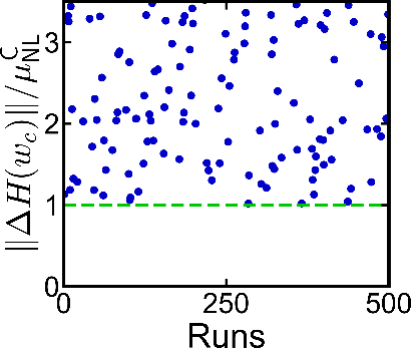
Topological protection of the existence of self-consistent nonlinear solutions. Each blue dot represents the potential strength ||*δH*|| where a solution curve of a nonlinear solution ceases to exist, while the green dashed line is the maximum of the local gap μ_NL_ within the induced topological phase change. Adapted with permission from ref 52. Copyright 2023 American Physical Society.

### 3.3 Numerical Efficiencies

Although the spectral localizer framework is applied to large finite systems with open boundaries, its formulas are ideally suited to leverage existing sparse matrix methods to yield efficient numerical implementations. In typical tight-binding bases, *H* is sparse, and the position operators are diagonal matrices that simply denote the coordinates of these vertices, with [*X*]_*j*,*j*_ = *x*_*j*_ and [*Y*]_*j*,*j*_ = *y*_*j*_ where the *j*th site is located at (*x*_*j*_, *y*_*j*_). As such, *L*_(***x***,*E*)_ is generally sparse, and the local gap can be calculated by using standard sparse eigenvalue solvers or sparse singular value solvers. Moreover, a matrix’s signature can be determined without calculating a single eigenvalue by instead using Sylvester’s law of inertia.^66^ In particular, if *L*_(***x***,*E*)_ = *NDN* ^†^ is the LDLT decomposition of the spectral localizer, with *N* lower triangular and *D* diagonal, then sig­[*L*_(***x***,*E*)_] = sig­[*D*], which is trivial to calculate given its structure.

## 4 Pseudospectra in Non-Hermitian Topological Lattices

### 4.1 Non-Hermitian Topological Photonic Lattices

In this section, we are going to discuss several novel aspects related to non-Hermitian topological lattices. Indeed non-Hermiticity provides an extra degree of freedom that offers many possibilities for designing new photonic platforms. Especially, in the context of non-Hermitian photonics,^67–74^ where complex Hamiltonians can be experimentally realized, a new direction has been established.^75^ One of the unique features of such open systems is the so-called exceptional points (EPs), where two or more eigenvalues and eigenvectors coalesce for a particular value of the systems parameter, thus forming a higher order exceptional point of *n*th-order (EP*n*).^75^ Recent impressive experiments reveal the physical impact of operating around the EP*n*’s, since ultra sensitive sensors^71^ and non-Hermitian gyroscopes,^72^ have been demonstrated.

Regarding the topological aspects of non-Hermitian photonics, some representative experiments of these are topological insulator lasers^76,77^ and the PT -symmetry breaking in a non-Hermitian Su-Schrieffer-Heeger (NHSSH) lattice, based on nonlinearity.^78^ It is generally true^79^ that many facts of topological insulators are not valid or they have to re-examined, when considering open systems. For example, one crucial question that arises based on the above discussion is the antagonistic relation between the extreme sensitivity on the one hand (due to non-Hermiticity) and the topological robustness on the other (due to topological robustness). In order to systematically investigate this question, a new mathematical framework is needed, that of pseudospectra theory.^80,81^ Pseudospectra are ideal for non-Hermitian systems, since they describe both the ultrasensitivity and the extreme power dynamics of the system and can provide critical information beyond the traditional eigenspectra approaches. Pseudospectra analysis of actual physical systems go beyond the context of fluid mechanics that were initially introduced,^80,81^ and they have been studied lately in the various non-Hermitian optical systems.^13,82–86^ Our analysis is general and can be applied to any non-Hermitian topological system, but still we consider a particular example relevant to the recent experiment of a NHSSH lattice that exhibits an EP3.^78^

Based on the paraxial coupled mode theory, the wave dynamics can be described as

11 $$i\frac{\partial \psi_{n}}{\partial \xi }+\underset{m}{\sum }H_{n,m}\psi_{m}=0$$

where ξ is the propagation distance for waveguides or time for cavities, ψ_*n*_ is the complex amplitude of the field’s envelope at the *n*th-channel, and *H*_*n*,*m*_ is the real space Hamiltonian elements. The corresponding right eigenvalue problem is *H*|*u*_*n*_^*R*^⟩ = λ_*n*_|*u*_*n*_^*R*^⟩, where the eigenvalues λ_*n*_ define the eigenspectrum (denoted as σ­(*H*)) of the lattice, which is generally complex.

### 4.2 Complex and Structured Pseudospectra

The pseudospectrum (or geometrical spectrum), which is a concept that is rarely used in photonics,^13,82,83,85^ makes the connection between the nonunitary dynamics of a dynamical (linear but open) system and non-Hermitian random matrix theory. More specifically, for a non-Hermitian Hamiltonian *H*, its eigenvalue spectrum σ­(*H*) does not sufficiently describe the wave dynamics. Instead, the associated pseudospectrum σ_*ε*_(*H*) provides us with a better picture of wave evolution in time or space. In particular, the pseudomodes are associated with the time dynamics, while the pseudoeigenvalues are associated with the sensitivity of the system to external perturbations. This means that in this pseudospectra framework, one can examine in a unified way both amplification and sensitivity. In the Hermitian limit, the eigenspectrum and the pseudospectrum are almost identical, whereas for non-Hermitian could be significantly different. In that context, one intuitive definition of the *ε*-pseudospectrum of a non-Hermitian matrix *H*, with spectrum σ­(*H*), is the union of all spectra of the matrices *H* + *E*_*j*_ for *s* different realizations of the perturbations of strength *ε*,

12 $$\sigma_{\varepsilon }\left(H\right)=\overset{s}{\underset{i=1,∥E_{i}∥<\varepsilon }{\cup }}\sigma \left(H+E_{i}\right)$$

where *E*_*j*_ are full complex random matrices (with respect to its matrix elements), and ∥··∥ is the matrix norm which is defined by $∥A∥=\underset{x\neq 0}{\sup}\frac{∥Ax∥}{∥x∥}$ .^80^ In practice, the perturbation matrices *E*_*j*_ are obtained from any full complex random matrices *E*_*j*_^′^ and normalized such that ∥*E*_*j*_∥ = *ε*′ < *ε*.

In order to elucidate these concepts, we consider the simplest possible example, that of a PT -symmetric matrix

$$H_{2\times 2}=i\left(\begin{array}{cc} ig & \kappa \\ \kappa & -ig \end{array}\right)$$ 13

In particular, this prototypical matrix describes two elements (cavities or waveguides), one with gain and one with loss, that are evanescently coupled with coupling strength κ. The eigenspectrum of this system is plotted in Figure 5(a) with cyan dots on the complex plane for three different values of the gain-loss amplitude *g*, given that κ = 1. The EP2 happens at *g* = 1, and such a PT -symmetric “atom” has been extensively studied both theoretically and experimentally.^75^ Notice that the matrix is multiplied by an *i*, and thus the eigenvalues on the imaginary axis correspond to a real eigenspectrum. Moreover, within the same plot in Figure 5(a), the three corresponding pseudospectra (yellow dots) have also been calculated. As we can clearly see, the size of pseudospectrum cloud is maximum at the EP2, where its scaling reveals the order of the EP. In particular, the size of the pseudospectrum can be quantitatively described by the pseudospectral radius ρ_*ε*_,^80^ which is defined here locally as

14 $$\rho_{\varepsilon }=\max_{z\in B}\left|z\right|$$

with the *z* belonging on the subset *B* ⊂ σ_ϵ_(*H*) of interest. Calculating the pseudospectral radius of a cloud center around an EPn for different values of *ε* will the show the radius is of the order of *ε* ^1/*n*^ for a very small perturbation strength, as is known from Lidskii perturbation theory of Jordan matrices.^13,84^ As such, if one can calculate the pseudospectral radius of the central cloud that corresponds to the EP2 for *g* = 1 in Figure 5(a) for different values of *ε*, the radius will behave like *ε* ^1/2^ for a small *ε*.

**Figure 5:**
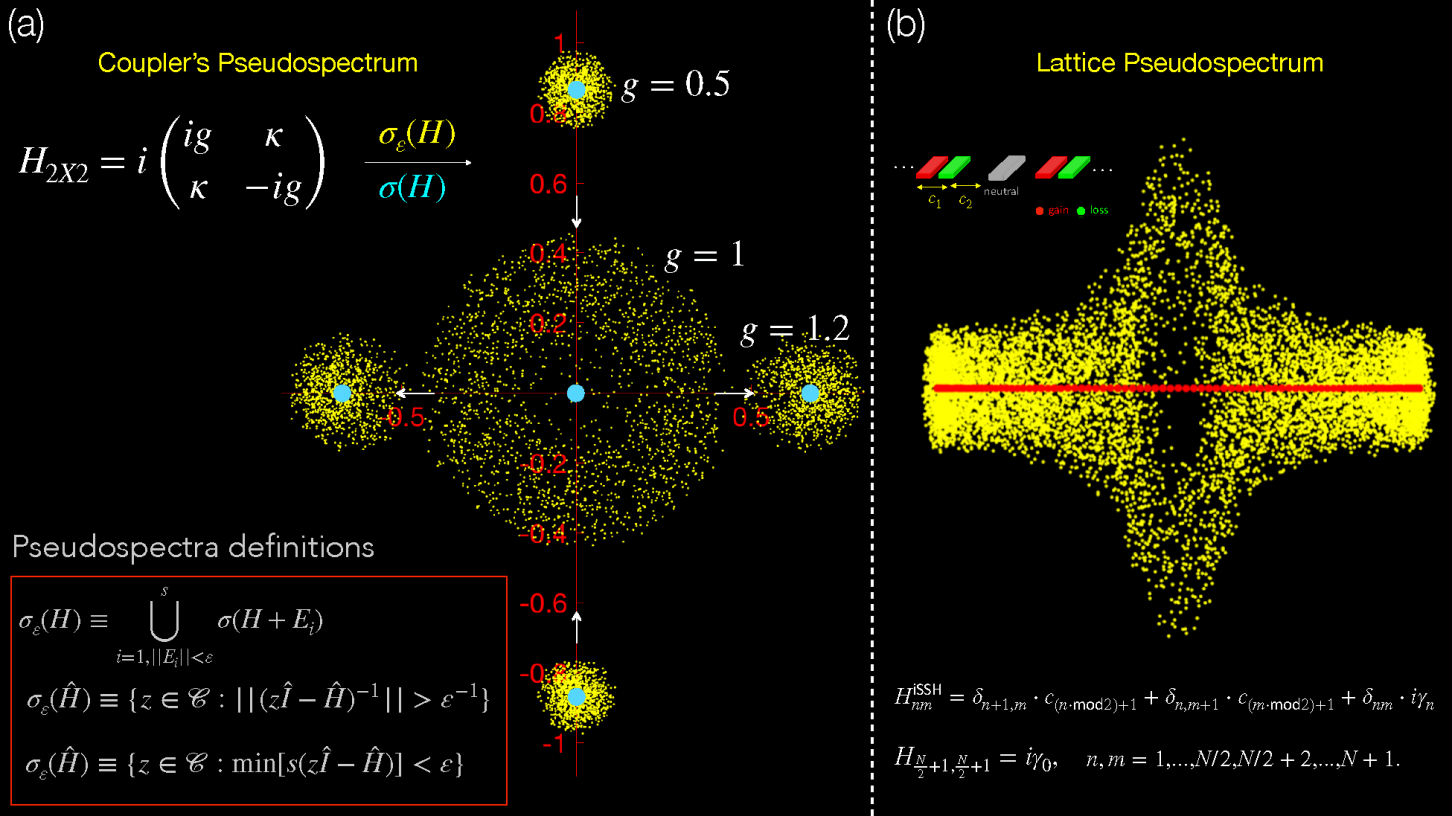
(a) Complex pseudospectra (yellow dots) on the complex plane of the simplest possible non-Hermitian system, the PT -coupler, for three different values of the gain-loss amplitude-*g* for κ = 1, before (*g* = 0.5), at (*g* = 1), and after (*g* = 1.2) the EP. (b) (Top left) Schematic depiction of the interface NH-SSH lattice and (top right) its corresponding complex pseudospectra (yellow dots). The formula of the matrix elements of the lattice is given on the bottom.

Now that we understand the meaning of the pseudospectrum, we move on to the NH-SSH lattice, which exhibits an EP3, and where the interplay between robustness and sensitivity is evident.^13,78^ More specifically, the lattice that we consider corresponds to two non-Hermitian SSH lattices coupled via a neutral element, as schematically illustrated in Figure 5(b). This extra channel at the interface has a tunable gain-loss amplitude γ_0_. Furthermore, the coupling constants are denoted by *c*_1_ and *c*_2_ for intra- and intercell coupling, respectively. The global gain-loss amplitude of each waveguide channel is described by the parameter γ, thus making the whole system non-Hermitian.

The associated complex pseudospectrum is plotted in Figure 5(b) on the complex plane (yellow dots), together with the eigenspectrum of the system (red dots). As in the two by two example of Figure 5, the pseudospectrum radius is maximum at the EP3. At this point, we note that the applied perturbations are complex and applied even in the zero entries of the Hamiltonian matrix *H*, meaning that these types of perturbations are of mathematical nature. Therefore, we examine the experimentally relevant structured perturbations, which are physically meaningful perturbations, and define the corresponding *structured pseudospectrum*.^80^ Namely, the structured pseudospectrum σ_*ε*_^*str*^ of the Hamiltonian *H*, is defined as

15 $$\sigma_{\varepsilon }^{str}\left(H\right)=\overset{s}{\underset{j=1,E-structured,∥E_{j}∥<\varepsilon }{\cup }}\sigma \left(H+E_{j}\right)$$

where *s* is the number of different realizations of the structured perturbations. Here we consider two types of structured perturbations, diagonal (*E*_*nm*_^′^ = δ_*n*,*m*_ *z*_*n*_) and off-diagonal (*E*_*nm*_^′^ = δ_*n*+1,*m*_ *z*_(*nmod*2)+1_ + δ_*n*,*m*+1_ *z*_(*mmod*2)+1_) perturbations, which are, respectively, perturbations on-site or on the coupling coefficients of the lattice. The real and the imaginary parts of the complex numbers *z*_*n*_ are drawn from the normal distribution around zero, and their magnitudes are on the order of one. What is important though is the value of *ε* that determines the physical strength of the applied perturbations. In Figure 6, the effect of the diagonal and off-diagonal perturbations is shown by calculating the corresponding structured pseudospectra before and at the EP3. More specifically, in Figure 6(a,b) we see that the structured (diagonal perturbations) pseudospectrum below the EP3 (the gap is still open) is exactly zero, due to topological robustness of the zero mode. On the other hand, exactly at the EP3, in Figure 6(c) we see the extended size of the structured pseudospectrum with a pseudosepctral radius is on the order of *ε* ^1/3^, for very small perturbation strength (Figure 6(d)).

**Figure 6:**
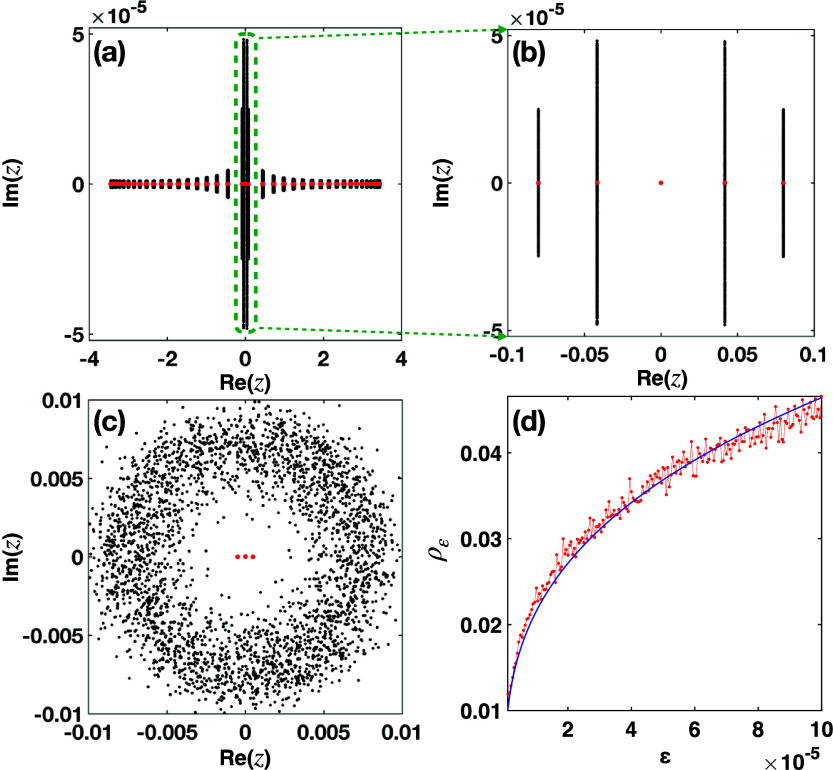
Structured pseudospectra $\sigma_{10^{-6}}^{str}\left(H\right)$ of the interface NHSSH lattice. (a) We include only chiral complex perturbations on the ±1 diagonals for *s* = 1000 realizations, and global gain/loss amplitude γ = 2.0155 (below the EP3). As we can see the zero mode indeed remains robust. (b) Magnified view of the selected area (green dashed line) which corresponds to the five modes closer to the origin of the complex plane. The size of the gap is ≈0.1. (c) Complex diagonal perturbations for *s* = 1000 realizations and γ = 2.0159293. (d) Pseudospectral radius (red line) of (c) as a function of *ε* ∈ [10^–6^, 10^–4^]. The blue line is ≈*ε* ^1/3^ and shown for comparison.

The fact that the zero-mode is robust to such off-diagonal/chiral perturbations for an open band gap is expected, but what happens exactly at EP3 for zero gap is a highly nontrivial problem. The answer is given by the structured pseudospectrum and reveals that the sensitivity scales similarly with that of an EP2. This means that even if the eigenspectrum is the same, the structured pseudospectrum is not. Thus, we arrive to the counterintuitive conclusion that when the system is exactly at the EP3, its sensitivity is that of EP3 for diagonal perturbations, but for off-diagonal perturbations is that of an EP2.^13^

At this point, we note that all previous discussions about pseudospectra are based on linear systems. Extending this approach to nonlinear optical problems in order to capture dynamic effects, such as mode coupling and energy transfer, is challenging and deserves further investigation. A first attempt is the study of ref 13 that treats the nonlinearity in an effective way as a defect at a particular propagation distance.

### 4.3 Future Directions

In this section, we reviewed the recent theoretical results based on pseudospectra theory, which highlight the fundamental question of the interplay between ultra sensitivity and topological protection and learned that the approach may provide insight for the study of other lattices of non-Hermitian topological physics. Methods beyond the traditional eigenspectra analysis are required to reveal the underlying characteristics of non-Hermitian sensitivity and dynamics in every topological open system.

Regarding the non-Hermitian systems, there are many open questions in the context of topological photonics. However, three different directions certainly deserve our attention: (a) topological insulator transport and pseudospectra, (b) nonlinear pseudospectra, and (c) the application of the notion of pseudospectra in Hermitian topological structures.

## 5 Thermodynamic Evolution of Nonlinear Topological Photonic Systems

The nonlinear propagation of topologically protected edge states is typically observed in the presence of high nonlinearities. However, the system’s behavior can become largely unpredictable when the conditions leading to this inherently self-focusing response are no longer met. Under weakly nonlinear conditions, a topological system may exhibit signs of chaos driven by a continuous exchange of power among all bulk and edge modes. In this regime, a traditional analysis via kinetic or dynamic approaches becomes challenging, particularly as the system’s size and dimensionality increase to hundreds of linear modes. Alternatively, information in these settings can be inferred statistically or thermodynamically.

### 5.1 Birth of Optical Thermodynamics

In recent years a new thermodynamic framework for light has been put forward, offering a powerful toolset to decipher the statistical response of nonlinear optical systems supporting many linear modes.^87,88^ This formalism establishes a comparison between the nonlinear dynamics of light and the thermal dynamics of gas particles in a contained environment. In a nonlinear setting, the power exchange of light among the linear modes adheres to strict conservation laws, akin to those governing the behavior of molecular gases, such as the conservation of total kinetic energy and particle number. Within the framework of optical thermodynamics, photonic systems typically exhibit two constants of motion: the total optical power *P* (a consequence of conservative evolution) and the linear part *U* of the total Hamiltonian energy. The second quantity (*U*) represents the effective “kinetic energy” of the system and acts as an effective quasi-invariant in the weakly nonlinear regime, where its fluctuations naturally diminish. In other words, under conditions of strong self-focusing or nonlinear localization, light will behave as a “photon gas”, where photons can interact across the entire physical space of a finite nonlinear optical environment while preserving their total “kinetic energy” and number. Accordingly, the two conservation laws of *P* and *U* will directly govern the thermal equilibrium state of this optical environment, allowing for a rigorous analysis through statistical mechanics and thermodynamics. As a result, a multimode optical configuration can be inherently characterized by effective thermodynamic variables, such as the optical temperature *T* and chemical potential μ, which correspond to actual thermodynamic forces that govern the “energy” and power exchange between nonlinear optical systems.

### 5.2 Rayleigh-Jeans Distribution and Optical Thermodynamic Parameters

The theory of optical thermodynamics is broadly applicable and can accurately describe and predict the statistical response of various multimode optical setups including optical cavities, multimode fibers, and waveguide lattices. In this respect, it is particularly useful for topological systems that can accommodate numerous nonlinear interacting modes. These modes correspond to the linear eigenstates ψ_*L*_^*i*^ of the Hamiltonian *H*, encompassing both edge and bulk states. Within the context of optical thermodynamics, the form of the nonlinear operator *H*_*NL*_ (i.e., Kerr, saturable-type, etc.) is inconsequential to the equilibrium conditions and is only responsible for the chaotic wave-mixing of power that leads to thermalization. Under conditions that preserve both the optical power *P* = *∑*_*i*_|*c*_*i*_|^2^ and the optical kinetic energy $U=-\underset{i}{\sum }E_{i}{\left|c_{i}\right|}^{2}$ (these definitions are universal, with *c*_*i*_ corresponding to the complex amplitude of the linear eigenstate ψ_*L*_^*i*^ with eigenvalues $E_{i}$ ), a weakly nonlinear system will equilibrate into a Rayleigh-Jeans (RJ) distribution, given by

16 $${\left|c_{i}\right|}^{2}=-\frac{T}{E_{i}+\mu }$$

where |*c*_*i*_|^2^ represents an ensemble averaged value of the modal occupancies, with *T* and μ corresponding to the optical temperature and chemical potential, respectively. The ensemble average described by eq 16 can be understood in two distinct yet equivalent ways: a RJ distribution may represent either the average of the modal occupancies measured at the output of the system across many realizations of a simulation/experiment or the time average occupancies of a single realization over a sufficiently long time interval.

The theory of optical thermodynamics marks an important milestone by establishing, for the first time, a rigorous framework for determining the optical temperature *T* and chemical potential μ directly from excitation conditions. It has been shown that any nonlinear multimode configuration evolving under the constancy of *U* and *P* will obey a universal equation of state, given by

17 U−μP=MT

associating the extensive variables (*U*, *M*, *P*), where *M* is the total number of modes, with the intensive variables (*T*, μ).^87^ To rigorously calculate the optical temperature and chemical potential and accurately predict the RJ occupancies, one needs to define and solve a system of two independent equations. The first is the equation of state (eq 17), while the second is determined by the equation for the optical power, after substituting the RJ occupancies from eq 16

18 $$P=\underset{i}{\sum }{\left|c_{i}\right|}^{2}=-\underset{i}{\sum }\frac{T}{E_{i}+\mu }$$

Substituting for μ from the equation of state, *U* – *μP* = *MT*, eq 18 becomes

19 $$P=\underset{i}{\sum }{\left|c_{i}\right|}^{2}=-\underset{i}{\sum }\frac{T}{E_{i}+\left(U-MT\right)/P}$$

Given the initial condition (*U*, *P*), eq 19 can be directly solved for *T*. While this equation has no known analytical solution for arbitrary spectra $E_{i}$ , it is a simple algebraic problem and trivial to solve numerically. It is important to realize that, while eq 19 yields multiple solutions for *T*, a physically allowed solution is unique, and hence *T* is unambiguously found for a given initial input. Once the temperature is determined, the associated μ can be derived in a straightforward manner from the equation of state.

The optical temperature of a nonlinear system reflects the tendency of light to concentrate, on average, toward the lower order modes, for positive temperatures, or the higher order modes, for negative temperatures, approaching over time the form of a RJ distribution. Recent experiments have demonstrated RJ equilibration in both the positive-temperature regime using optical fibers and the negative-temperature regime in nonlinear fiber loop lattices.^16^ This broad applicability of RJ thermalization is evident even in systems exhibiting unconventional properties. It has been shown, for example, that even disordered optical lattices, where the eigenstates become heavily localized, will attain the theoretically predicted RJ equilibria following a very slow process of optical thermalization. Meanwhile, the OAM systems, characterized by an additional conservation law (that of orbital angular momentum), will also attain thermalization following the RJ law. The same rules apply to non-Hermitian and topological configurations, where novel thermal phenomena can manifest.

### 5.3 Thermalization in Nonlinear Optical Topological Structures

In topological settings, given that a statistical distribution is expressed in the eigenmode basis—where all topological properties are defined—one can directly monitor the interplay between the topological edge flow and the thermalization process.^89^ In general, under weakly nonlinear conditions, the formation of nonlinear modes or soliton structures is unsustainable, leading to power leakage into the bulk and the breakdown of topological protection, even if the initial excitation is confined to the lattice edge. Nonetheless, thermal equilibrium can eventually be reached. Given the specific input conditions, one can accurately employ the methodology outlined above to predict the average power occupancies for both bulk and edge states and consequently the average power ratio between them. These new theoretical tools thus open up new possibilities for thermally controlling the topological edge flow by identifying optimal initial conditions that maximize power retention within the edge states.

Interestingly, the underlying topology of a nonlinear lattice gives rise to distinct thermalization dynamics, depending on the topological invariant of its linear band structure. For example, in a two-band Chern insulator, a trivial Chern number *C* = 0 favors prethermalization into two separate RJ distributions, each corresponding to the upper and lower sub-band groups. The large band gap separating the groups effectively prevents power exchange between them while still allowing the exchange of the Hamiltonian’s internal energy *U*. Consequently, the two RJ nonthermal equilibria will share the same optical temperature *T*_*u*_ = *T*_*d*_ (for the ‘up’ and ‘down’ sub-bands) but have different chemical potentials μ_*u*_ and μ_*d*_. These states are classified as prethermal, recognizing that the nonlinear wave-mixing process will eventually drive the system toward a true global equilibrium. Interestingly, both the global equilibrium and the prethermal RJ states can be predicted from initial conditions by considering only the eigenvalues of the two sub-bands separately in eq 18. Conversely, when the system enters its nontrivial topological regime, the edge states bridge the band gap, facilitating rapid power flow between the sub-band and accelerating thermalization into a global RJ distribution, bypassing the prethermalization stage.

## 6 Conclusions and Outlook

In summary, over the past decade, several novel theoretical frameworks have been developed to classify the topological phases of nonlinear topological insulators. Among these, machine learning has emerged as a particularly promising tool for exploring large, highly dimensional parameter spaces. This approach enables the identification of dynamic phases and offers insights into the stability of modes that evolve over time, which are often difficult to capture by using traditional techniques. In contrast, within the frequency domain, the spectral localizer framework has proven to be a powerful method for computing local Chern markers that characterize topological protection based on position-dependent spectral information. This framework not only bridges the gap between the topological descriptions of fully periodic systems and finite-sized structures but also makes it possible to precisely determine topological phase transitions in nonlinear systems under localized excitation. Moreover, the pseudospectra method provides a complementary perspective by allowing researchers to evaluate the robustness and stability of exceptional points in non-Hermitian topological systems. This approach offers valuable physical insight into the resilience of topologically protected modes under perturbations. Lastly, a thermodynamic framework has been introduced for describing nonlinear multimode waveguides in which macroscopic parameters derived from the Rayleigh–Jeans distribution can be used to characterize thermodynamic evolution processes such as optical thermalization. Together, these diverse approaches contribute significantly to advancing our understanding of nonlinear topological photonics and open new pathways for future exploration.

Overall, recent advances in the theoretical understanding of nonlinear topological photonic insulators have introduced accessible and insightful parameters that capture the distinctive features of nonlinear coupled optical waveguide systems. These include the temporal stability of dynamic modes, the topological protection associated with local spectral gaps, the robustness of exceptional points, and the behavior of many-photon dynamics. We believe that these developments not only deepen the theoretical insight into nonlinear topological optical modes but also provide powerful tools for exploring nonlinear effects in a wide range of coupled optical resonator systems.
